# Show me the data

**Published:** 2008-02

**Authors:** Mike Rossner, Heather Van Epps, --- Emma Hill

**Affiliations:** Executive Director, the Rockefeller University Press; Executive Editor, the Journal of Experimental Medicine; Executive Editor, the Journal of Cell Biology

The integrity of data, and transparency about their acquisition, are vital to science. The impact factor data that are gathered and sold by Thomson Scientific (formerly the Institute of Scientific Information, or ISI) have a strong influence on the scientific community, affecting decisions on where to publish, whom to promote or hire,^1^ the success of grant applications,^2^ and even salary bonuses.^3^ Yet, members of the community seem to have little understanding of how impact factors are determined, and, to our knowledge, no one has independently audited the underlying data to validate their reliability.

## Calculations and negotiations

The impact factor for a journal in a particular year is declared to be a measure of the average number of times a paper published in the previous two years was cited during the year in question. For example, the 2006 impact factor is the average number of times a paper published in 2004 or 2005 was cited in 2006. There are, however, some quirks about impact factor calculations that have been pointed out by others (e.g. references 1, 4, 5), but which we think are worth reiterating here:

● The numerator of the impact factor contains every detectable citation to a journal’s content from the previous two years, regardless of the article type.^6^ For example, the 2006 impact factor numerator contains all citations to all content published in 2004 and 2005. The denominator of the impact factor, however, contains only those articles designated by Thomson Scientific as primary research articles or review articles. Journal ‘front matter’, such as *Nature* ‘News and Views’ is not counted.^4^ Thus, the impact factor calculation contains citation values in the numerator for which there is no corresponding value in the denominator.
● Articles are designated as primary, review, or ‘front matter’ by hand by Thomson Scientific employees examining journals^6^ using various bibliographic criteria, such as keywords and number of references.^7^
● Some publishers negotiate with Thomson Scientific to change these designations in their favour.^5^ The specifics of these negotiations are not available to the public, but one can’t help but wonder what has occurred when a journal experiences a sudden jump in impact factor. For example, Current Biology had an impact factor of 7.00 in 2002 and 11.91 in 2003. The denominator somehow dropped from 1 032 in 2002 to 634 in 2003, even though the overall number of articles published in the journal increased (see ISI Web of Science: http://portal.isiknowledge.com/, subscription required).
● Citations to retracted articles are counted in the impact factor calculation.^8^ In a particularly egregious example, Woo Suk Hwang’s stem cell papers in Science from 2004 and 2005, both subsequently retracted, have been cited a total of 419 times (as of November 20, 2007). We won’t cite them again here to prevent the creation of even more citations to this work.
● Because the impact factor calculation is a mean, it can be badly skewed by a ‘blockbuster’ paper. For example, the initial human genome paper in *Nature*^9^ has been cited a total of 5 904 times (as of November 20, 2007). In a self-analysis of their 2005 impact factor, Nature noted that 89% of their citations came from only 25% of the papers published.^4^
When we asked Thomson Scientific if they would consider providing a median calculation in addition to the mean they already publish, they replied, ‘It’s an interesting suggestion … The median … would typically be much lower than the mean. There are other statistical measures to describe the nature of the citation frequency distribution skewness, but the median is probably not the right choice.’Perhaps so, but it can’t hurt to provide the community with measures other than the mean, which, by Thomson Scientific’s own admission, is a poor reflection of the average number of citations gleaned by most papers.
● There are ways of playing the impact factor game, known very well by all journal editors, but played by only some of them. For example, review articles typically garner many citations, as do genome or other ‘data-heavy’ articles (see example above). When asked if they would be willing to provide a calculation for primary research papers only, Thomson Scientific did not respond.

## Integrity

As journal editors, data integrity means that data presented to the public accurately reflect what was actually observed. To help ensure this, the Rockefeller University Press instituted a policy of scrutinising image data in accepted manuscripts for evidence of manipulation. We realise that image data is only one type of data we publish, but it is a type that can easily be examined for integrity. If a question is raised about the data in a figure, we ask the authors to submit the original data for examination by the editors. We consider it our obligation to protect the published record in this way.

Thomson Scientific makes its data for individual journals available for purchase. With the aim of dissecting the data to determine which topics were being highly cited and which were not, we decided to buy the data for our three journals (the *Journal of Experimental Medicine, the Journal of Cell Biology, and the Journal of General Physiology*) and for some of our direct competitor journals. Our intention was not to question the integrity of their data.

When we examined the data in the Thomson Scientific database, two things quickly became evident: first, there were numerous incorrect article-type designations. Many articles that we consider ‘front matter’ were included in the denominator. This was true for all the journals we examined. Second, the numbers did not add up. The total number of citations for each journal was substantially fewer than the number published on the Thomson Scientific, Journal Citation Reports (JCR) website (http://portal.isiknowledge.com, subscription required). The difference in citation numbers was as high as 19% for a given journal, and the impact factor rankings of several journals were affected when the calculation was done using the purchased data (data not shown due to restrictions of the license agreement with Thomson Scientific).

## Your database or mine?

When queried about the discrepancy, Thomson Scientific explained that they have two separate databases − one for their ‘Research Group’ and one used for the published impact factors (the JCR). We had been sold the database from the ‘Research Group’, which has fewer citations in it because the data have been vetted for erroneous records. ‘The JCR staff matches citations to journal titles, whereas the Research Services Group matches citations to individual articles’, explained a Thomson Scientific representative. ‘Because some cited references are in error in terms of volume or page number, name of first author, and other data, these are missed by the Research Services Group.’

When we requested the database used to calculate the published impact factors (including the erroneous records), Thomson Scientific sent us a second database. But these data still did not match the published impact factor data. This database appeared to have been assembled in an *ad hoc* manner to create a facsimile of the published data that might appease us. It did not.

## Opaque data

It became clear that Thomson Scientific could not or (for some as yet unexplained reason) would not sell us the data used to calculate their published impact factor. If an author is unable to produce original data to verify a figure in one of our papers, we revoke the acceptance of the paper. We hope this account will convince some scientists and funding organisations to revoke their acceptance of impact factors as an accurate representation of the quality − or impact − of a paper published in a given journal.

Just as scientists would not accept the findings in a scientific paper without seeing the primary data, so should they not rely on Thomson Scientific’s impact factor, which is based on hidden data. As more publication and citation data become available to the public through services like PubMed, PubMed Central, and Google Scholar®, we hope that people will begin to develop their own metrics for assessing scientific quality rather than rely on an ill-defined and manifestly unscientific number.

## References

[1] Monastersky R (2005). The number that’s devouring science. The impact factor, once a simple way to rank scientific journals, has become an unyielding yardstick for hiring, tenure, and grants.. Chron High Educ.

[2] Wells WA (2007). The returning tide: how China, the world’s most populous country, is building a competitive research base.. J Cell Biol.

[3] (2006). Editorial. Cash-per-publication is an idea best avoided.. Nature.

[4] (2005). Editorial. Not-so-deep impact. Research assessment rests too heavily on the inflated status of the impact factor.. Nature.

[5] (2006). The PLoS Medicine Editors. The impact factor game. It is time to find a better way to assess the scientific literature.. PLoS Med.

[6] Garfield E (1999). Journal impact factor: a brief review.. Can Med Assoc J.

[7] The Thomson Scientific Impact Factor 1994.. http://scientific.thomson.com/free/essays/journalcitationreports/impactfactor/.

[8] Liu SV (2007). Hwang’s retracted publication still contributes to Science’s impact factor.. Sci Ethics.

[9] Lander ES, Linton LM, Birren B, Nusbaum C, Zody MC, Baldwin J (2001). Initial sequencing and analysis of the human genome.. Nature.

